# Validation of the Simple Shoulder Test in a Portuguese-Brazilian Population. Is the Latent Variable Structure and Validation of the Simple Shoulder Test Stable across Cultures?

**DOI:** 10.1371/journal.pone.0062890

**Published:** 2013-05-13

**Authors:** Jose Osni Bruggemann Neto, Rafael Lehmkuhl Gesser, Valdir Steglich, Ana Paula Bonilauri Ferreira, Mihir Gandhi, João Ricardo Nickenig Vissoci, Ricardo Pietrobon

**Affiliations:** 1 Orthopedics and Traumatology Institute (IOT), Joinville, Santa Catarina, Brazil; 2 University of Joinville Region (UNIVILLE), Dentistry Department, Joinville, Santa Catarina, Brazil; 3 Research on Research Group, Duke University Health System, Durham, North Carolina, United States of America; 4 Duke-NUS Graduate Medical School, Singapore, Singapore; 5 Singapore Clinical Research Institute, Singapore, Singapore; 6 Faculdade Inga, Medicine Department, Maringá, Paraná, Brazil; 7 Department of Surgery, Duke University Health System, Durham, North Carolina, United States of America; Tokyo Metropolitan Institute of Medical Science, Japan

## Abstract

**Background:**

The validation of widely used scales facilitates the comparison across international patient samples. The objective of this study was to translate, culturally adapt and validate the Simple Shoulder Test into Brazilian Portuguese. Also we test the stability of factor analysis across different cultures.

**Objective:**

The objective of this study was to translate, culturally adapt and validate the Simple Shoulder Test into Brazilian Portuguese. Also we test the stability of factor analysis across different cultures.

**Methods:**

The Simple Shoulder Test was translated from English into Brazilian Portuguese, translated back into English, and evaluated for accuracy by an expert committee. It was then administered to 100 patients with shoulder conditions. Psychometric properties were analyzed including factor analysis, internal reliability, test-retest reliability at seven days, and construct validity in relation to the Short Form 36 health survey (SF-36).

**Results:**

Factor analysis demonstrated a three factor solution. Cronbach’s alpha was 0.82. Test-retest reliability index as measured by intra-class correlation coefficient (ICC) was 0.84. Associations were observed in the hypothesized direction with all subscales of SF-36 questionnaire.

**Conclusion:**

The Simple Shoulder Test translation and cultural adaptation to Brazilian-Portuguese demonstrated adequate factor structure, internal reliability, and validity, ultimately allowing for its use in the comparison with international patient samples.

## Introduction

Shoulder pain is the second more common musculoskeletal disorder in the primary care setting [1], representing more than 6 million orthopedic visits a year in the U.S [2]. Of 1000 patients who visit a clinic, 35 have shoulder pain, and of these 60% are women [3]. It is estimated that 20% of people will suffer from shoulder pain at some point in their lives [1]. Shoulder pathologies may cause pain, leading to reduced joint mobility and therefore have an impact on quality of life of individuals [4]. Its influence on productivity and total number of worked hours has a major socio-economic impact [5], [6].

Conditions affecting the shoulder should be evaluated based on how activities of daily living might be affected. One of the many evaluating instruments is the Simple Shoulder Test, which has not yet been translated and validated to Brazilian-Portuguese. Also, little is known about its content structure since there are very few publications addressing it [7], [8]. Considering Brazilian population size, ethnic and geographical diversity the availability of a translated and validated version of this scale would allow international researchers to reach this huge number of subjects what could ease the development of not only cross-cultural studies, but also studies about rare manifestations or diseases of the shoulder. It would also help evaluating the content structure of this specific scale, since there are few publications addressing it.

The Simple Shoulder Test scale is a tool designed to evaluate functional limitations of an injured shoulder that compromise an individual’s daily activities [9]. It is a practical method for assessment of shoulder function before and after treatment [10]. The questionnaire was developed based on common patient complaints presented to practitioners. Numerous studies have used this test for the assessment of shoulder function [11], [12], implying its importance and strengthening the need for cultural translation and validation to other languages, facilitating assessment of shoulder conditions among different populations groups. Test-retest reliability, construct validity and responsiveness of the SST (Simple Shoulder Test) have been thoroughly studied [11], [7], [13], [14], [15]. This questionnaire is widely used since the ease of its application has facilitated the comparison of patient outcomes. It is also a helpful indicator of the time required to reach a maximum benefit of a treatment for shoulder pain.

The objective of this study was to translate, adapt culturally, and validate the Simple Shoulder Test to Brazilian Portuguese. Also we tested the stability of factor analysis across different cultures.

## Methods

### Ethics

Approval was obtained from the Research Ethics Committee of Hospital Municipal São José prior to the initiation of this project. All study participants provided written informed consent prior to enrollment in the study.

### Participants

A total of 100 patients from Hospital Municipal São José (Joinville, SC) underwent physical examination and those with a diagnosis of shoulder conditions such as rotator cuff tears, inflammation and degenerative arthritis were enrolled in the study. Patients were asked about their limitations in terms of shoulder function, and recruited only when a functional limitation was present. No imaging evaluation was performed for research purposes.

### Simple Shoulder Test

Simple Shoulder Test is a standardized instrument developed to systematically document shoulder function. This questionnaire consists of 12 questions with "yes" or "no" answers about the function of the affected shoulder (Appendix S1). Answers to these questions provide a standardized way of recording shoulder function before and after treatment. It also provides a functional assessment of the outcome of a specific treatment for certain conditions of the shoulder [16], [17].

Simple Shoulder Test’s scores for each dimension are calculated through the mean of the alternative responses for each question, following the formula: Total Score = Σy/x, where y = answer for each question in the dimension and x = number of questions for that dimension. This calculation results in a value within the range of the Likert scale of the test for all subscales regardless of the number of items in each subscale.

### SF-36

The Brazilian version of the SF-36 is a self-administered generic health status measure with 36 items. It measures three major health attributes, namely functional status, well-being, overall health. These items are also grouped with eight subscales, namely physical function, role limitations due to physical health, bodily pain, general health, vitality, social function, role limitations due to emotional health, and mental health [18].

### Initial Translation into the Brazilian Portuguese Language

Three bilingual translators [JB, RG, RP] whose native language is Brazilian Portuguese translated the questionnaire from English into Brazilian Portuguese. Two of the translators [JB, RP] were aware of the concepts on the questionnaire. The third translator [RP] was neither aware of nor informed about the conceptual content. All translators had expertise in cross-cultural translation scale study design and are fluent in both Brazilian Portuguese and English.

### Translation Synthesis

This stage consisted of the synthesis of all three translations. A fourth person [APF], who also has previous experience in scale validation, had the role mediating the discussions related to the divergences in translations. This synthesis process was fully documented. All disagreements were resolved through discussion, ultimately reaching a consensus.

### Back Translation to English

Two bilingual translators [JS, MM], whose native language was English, translated the synthesized version back into English. The purpose of this back-translation was to ensure that the original content of the questionnaire had been reliably translated, with no major deviations.

### Expert Committee

The committee was constituted by one physician [RF] and all six translators. The translation synthesis and back-translation versions of the Simple Shoulder Test were submitted to the expert committee, which reviewed all translations and attempted to reach a consensus regarding differences identified in the process. A pre-final version of the Brazilian-Portuguese translation of the questionnaire was developed and pre-tested. The main guiding principle was that the final test should make it easy for an ordinary individual to understand it.

### Qualitative Evaluation

The pre-final version of the Simple Shoulder Test questionnaire was administered in a group of twenty patients who had an appointment at the orthopedic ambulatory because of shoulder pathologies. This phase was aimed at certifying whether the patients understood the meaning of the questions present in the Simple Shoulder Test questionnaire. Based on the observations, the expert committee modified and prepared a final version of the Simple Shoulder Test questionnaire in Brazilian-Portuguese.

### Test of Final Version

The translated version of the questionnaire [Appendix S1] was administered to 100 patients [19], [20] with a diagnosis of shoulder conditions such as rotator cuff tears, inflammatory processes, and degenerative arthritis evaluated at the orthopedic clinic of the Hospital Municipal São José, in Joinville, Brazil. We did not impose restrictions on age or gender. After one week, 20 patients were randomly selected to participate in the test-retest reliability section of our study [21]. Random sample schedules were generated through a list of pseudo-random numbers using the R Language [22].

### Evaluation of Psychometric Properties

Factor structure of the Simple Shoulder Test translated version to Brazilian-Portuguese was analyzed using exploratory factor analysis with oblique and orthogonal rotations and confirmatory factor analysis. The internal consistency of the Simple Shoulder Test translated version to Brazilian-Portuguese was examined using Cronbach’s alpha. Alpha values >0.70 were deemed acceptable [23].

Test-retest reliability, measured by the intra-class correlation coefficient (ICC), was analyzed at seven days (n = 20). ICC can vary from 0.00 to 1.00, where values of 0.60 to 0.80 are considered as good reliability and with those above 0.80 indicating excellent reliability [24].

Validity, or the ability of the scale to measure what it is intended to measure, was also evaluated by measuring the correlation between scores from the Simple Shoulder Test and the Short-form Health Survey (SF-36). Research has demonstrated that the SF-36 is highly reliable and responsive to global quality of life measurements in patients with musculoskeletal and shoulder disorders [25].

### Statistical Methods

Statistical analyses were performed using Stata/SE software (version 9.0) for Windows (Stata, College Station, Texas) and the R Language (R Core Team 2012) [22]. Factor analysis was performed through the “mirt” [26] and “sem” [27] R packages. Initially, descriptive analyses employing means and percentages with 95% confidence intervals were used to establish the sample’s demographic and clinical characteristics. We used the Mahalobis distance to identify univariate and multivariate outliers [28].

Latent structure was tested through exploratory and confirmatory factor analysis. Appropriate number of factors to retain was established analysing the scree test [29], eigen-values (EV) (above 1.0), root mean square standard errors (residuals, less than 0.05) and cumulative variance explained by the factor structure and factor interpretability.

Rotation was performed through both orthogonal (varimax) and oblique (promax) methods, using a multidimensional item response theory model. We only reported results for promax rotation because the Simple Shoulder Test questions were correlated. CFA was performed based on a polychoric correlation matrix, as suggested in the sem package. The following model fit indexes was used to test and assess hypothesized model adequacy, parsimony and fitness in confirmatory factor analysis: chi-square, Root Mean Square Error of Aproximation (RMSEA) (values inferior to 0.05 are considered as adequate fit) [30]; Comparative Fit Index (CFI) (values superior to 0.95 are accepted as good fit) [31]; Goofness-of-fit Index and Adjusted Goodness of fit Index (GFI/AGFI) (values superior to 0.90 are interpreted as acceptable fit) [32]; Tucker-Lewis Index (TLI) (acceptable fit with values superior to 0.97) [33] and Akaike Information Criteria/Bayesian Information Criteria (AIC/BIC) (lower values indicate better model when compared to other models) [33].

Comparison between latent construct structures was obtained by testing different factor structures and comparing the indicators reported in previous literature with the fit indicators found in the Brazilian sample.

Internal consistency was measured through Cronbach’s alpha coefficient. Intra-class correlation coefficient (ICC) was used to examine the test-retest reliability of the scale. Instrument construct validity was determined by the use of Spearman correlation coefficient between the Simple Shoulder Test and the SF-36.

## Results

### Baseline Characteristics

Most of the participants were female (n = 63, 63.0%), Caucasian (n = 98, 98.0%) and married (n = 67, 67.0%) (Table 1).

**Table 1:** Baseline participant demographics.

DEMOGRAPHICS	N = 100 (%)
**AGE (mean ± sd)**	45±1.41
**GENDER**	
Female	63 (63%)
**RACE**	
Caucasian	89 (89%)
Black	9 (9%)
Other	2 (2%)
**STATUS**	
Married	67 (67%)
Single	11 (11%)
Widow	10 (10%)
Separated	12 (12%)
**EDUCATION**	
Incomplete Basic School	36 (36%)
Basic School	17 (17%)
Incomplete High School	23 (23%)
High School	22 (22%)
Graduate School	2 (2%)

### SST’s Latent Variable Structure and Comparison with Reported Estimates

The SST’s latent variables structure has demonstrated instability in reported indicators regarding the number of constituting factors, and it was initially developed to assess only one latent structure (Shoulder Function). But when reevaluated it presented the possibility of representing a factorial structure of 2 factors (Table 2). The few number of publications reporting factor analysis or other latent models methods in cross-cultural validation made the analysis of the factor structure stability more difficult.

**Table 2:** Indicators of factor analysis of SST reported in previous literature.

Reported Cultures Validations	EFA			CFA
	Eigen Value	Variance Explained	Dimensions/Questions	
Lithuanian(Rylyskis et al., 2008)	NA	NA	NA	NA
Dutch(Van Kampen et al., 2012)	NA	NA	1 Dimension. Factors withlow loadings Q2 (0.39) andQ12 (0.43)	A 1-factor model fitted the data moderately. The CFI was 0.943, TLI was 0.931, and RMSEA was 0.068. Items 1, 2, and 12 had relatively low factor loadings
Roddey et al., 2000*	F1 4.84F2 1.46	52.6%	CL - Q8, Q10F1 - “Can do”F2 - “Comfort”	NA
Italian(Marchese et al.et al., 2012)	NA	NA	NA	NA

* This is not a cultural validation study but it evaluated the construct validity of SST scores through factor analysis.

EFA = Exploratory Factor Analysis; CFA = Confirmatory Factor Analysis; NA = not available; Q2 = question 2; Q12 = question 12; Q8 = question 8; Q10 = question 10; CFI = Comparative Fit Index; TLI = Tucker-Lewis Index; CL = cross loading; RMSEA = Root Mean Square Error of Aproximation; F1 = one factor; F2 = two factors.

In the Dutch version, when performing confirmatory factor analysis (CFA) the authors noticed indications of problems in the factor structure related to fit indicators and to dimensions with low loadings. Although the factor structure could be accepted, it was not preceded by an initial exploratory factor analysis (EFA) to verify the latent structure of the test.

Thus, to test the latent structure of SST, we determined the best factor structure for the Brazilian version of SST (SST-BR) and performed CFA against data gathered from other structures already reported in the literature (Table 2).

### Factor Analysis of the SST-BR

Initial exploratory data analysis using box plots and Mahalanobis distance showed neither missing values nor univariate and multivariate outliers. Exploratory factor analysis was then performed to identify the possible latent constructs beneath the set of responses (Table 3).

**Table 3:** Exploratory factor analysis results for simple shoulder test’s validations to Brazilian-Portuguese.

		1 Factor Solution	3 Factors Solution		
	EV				
Q1 - Is your shoulder comfortable with your arm at rest byyour side?	3.32	0.27			0.40
Q2 - Does your shoulder allow you to sleep comfortably?	1.61	0.18			0.37
Q3 - Can you reach the small of your back to tuck in yourshirt with your hand?	1.30	0.56			0.57
Q4 - Can you place your hand behind your head with theelbow straight out to the side?	1.07	0.48			0.43
Q5 - Can you place a coin on a shelf at the level of your shoulder without bending your elbow?	0.90	0.60	0.61		
Q6 - Can you lift one pound (a full pint container) to thelevel of your shoulder without bending your elbow?	0.81	0.57	0.96		
Q7 - Can you lift eight pounds (a full gallon container) tothe level of your shoulder without bending your elbow?	0.73	0.52	0.39		
Q8 - Can you carry twenty pounds at your side with theaffected extremity?	0.59	0.54		0.85	
Q9 - Do you think you can toss a softball under-handtwenty yards with the affected extremity?	0.51	0.25	0.48		
Q10 - Do you think you can toss a softball over-handtwenty yards with the affected extremity?	0.47	0.61		0.70	
Q11 - Can you wash the back of your opposite shoulderwith the affected extremity?	0.34	0.41	0.41		
Q12 - Would your shoulder allow you to work full-time atyour regular job?	0.29	0.33		0.35	
Variance Explained		0.22	0.15	0.28	0.38
RMSE		0.08	0.04		

EV = Eigen Values; RMSE = Root mean square error.

The criteria used to determine the number of factors to be retained suggested possible models from 1 to 3 factors, thus, 1, 2 and 3 factors solutions were tested and analyzed. Eigen-values varied between 3.32 and 0.29 (Table 3), with four values above 1. These values associated to the root mean square of the residuals of 0.05 and 0.04 indicated a good estimate for a 2 or 3-factors solution, respectively, differing from the original test structure. Also, the amount of variance explained by the 1 factor model was only 22%, while the 2 and 3-factors solution explained 32% and 38%, respectively. Nevertheless, a 2-factor solution was discarded during our interpretation since it indicated four observed variables (questions) with factor loadings lower than 0.35 (Table 3). The same problem was observed with the 1-factor solution. Since the Simple Shoulder Test is a scale with twelve items, it was chosen the structure with less exclusion of items.

Thus, a 3-factor solution was considered more adequate considering our criteria for factor structure (amount of variance explained, commonalities, factor loading analysis, eigen values, RSME) and clinical relevance. All factors demonstrated a satisfactory loading (above 0.40) and no cross-loadings, except Q2 and Q12 with loadings above 0.35 (Table 3).

**Figure 1:**
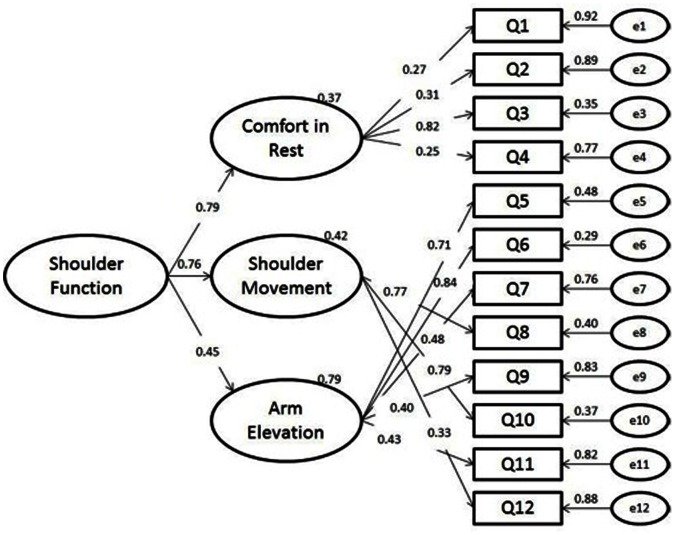
Confirmatory Factor Analysis.

The hypothesized model constituted by 3 latent variables and defined by 12 observed continuous variables, was then tested through Confirmatory Factor Analysis. However, as a way to clarify the difference from the original Simple Shoulder Test’s factor structure, a 1-factor model was also tested and compared to the adopted model (Table 4). First, the original structure of the scale was analyzed and poor fit indicators were found (RMSEA = 0.117; GFI = 0.810; AGFI = 0.726; CFI = 0.654; TLI = 0.577; AIC/BIC = 175.82/238.35). Observing the standardized factor weights for Model A, it was noticed that three paths were not significant and had loadings lower then 0.25. Therefore, a second model was developed for 1 factor solution, but with the exclusion of the items with low factor loadings. Unfortunately, fit indicators were not good enough (RMSEA = 0.129; GFI = 0.809; AGFI = 0.714; CFI = 0.655; TLI = 0.568; AIC/BIC = 160.63/217.95). The Confirmatory Factor Analysis for 3 factors structure model showed adequate fit indicators (Table 4) as well as lower levels of AIC/BIC when compared to the previous models (RMSEA = 0.029; GFI = 0.923; AGFI = 0.875; CFI = 0.980; TLI = 0.973; AIC/BIC = 112.09/190.25). Moreover, since SST has not shown a stable latent structure in the literature, the one variable latent construct of the original test was tested as a second order factor (Figure 1), showing that in a first order, when analyzing at the item level the SST showed 3 latent constructs. But when we analyzed the structure at the dimension level, the test loaded into one single latent construct, therefore, related to the original scale. The second order model was considered acceptable since it exposed similar fit indicators as the 3 factors model.

**Table 4:** Confirmatory factor analysis’ models fit indicators comparison.

Model Comparison	Description	X2 (Df)	RMSEA	GFI	AGFI	CFI	TLI	AIC/BIC
A	1 Factor	127.82 (54)	0.117	0.810	0.726	0.654	0.577	175.82/238.35
B	1 Factor - reducedquestions	116.63 (44)	0.129	0.809	0.714	0.655	0.568	160.63/217.95
C	2 Factors	127.14(53)	0.118	0.811	0.723	0.652	0.567	177.14/242.27
D	3 Factor	52.092 (66)	0.029	0.923	0.875	0.980	0.973	112.09/190.25
E	3 Factors in 1st Orderand 1 Factor in 2nd Order	58.994 (49)	0.045	0.914	0.864	0.953	0.936	116.99/192.54

Df = Degree of Freedom; RMSEA = Root Mean Square Error of Aproximation; GFI = Goofness-of-fit Index; AGFI = Adjusted Goodness of fit Index; CFI = Comparative Fit Index; TLI = Tucker-Lewis Index; AIC/BIC = Akaike Information Criteria/Bayesian Information Criteria.

All paths for the Brazilian-Portuguese Simple Shoulder Test’s confirmatory factor analysis model were statistically significant although some of the standardized factor weights demonstrated low values (Q1 = 0.27; Q2 = 0.31 and Q3 = 0.24) (Figure 1). Residual analysis did not indicate problems, with values raging from - 0.270 (1st quartile) and 0.592 (3rd quartile).

Three well-defined factors were developed, one related to *Arm Elevation*, one concerning *Shoulder Movement* and another concerning *Comfort with the Shoulder in Rest Positions*. In this process, shoulder function was considered a multi-factorial variable with a Global Value (GV) for general construct evaluation.

Results from confirmatory factor analysis (Table 4) did not demonstrate adequate fit indicators for psychometric properties for one factor structure (Models A e B). When comparing to the two factors model (Model C) reported in the literature, it also did not show a moderate fit.

### Psychometric Characteristics of the Simple Shoulder Test

#### Internal consistency

Internal consistency reports two estimates: Cronbach’s alpha and Guttman’s Lambda 6 (G6). Cronbach’s alpha was 0.82 (CI95% of 0.76 to 0.86) for overall test. 0.82 for *Arm Elevation* and *Shoulder Movement* subscales; 0.81 for *Comfort in Rest* subscale and 0.59 for Global Shoulder Function value. G6 reliability indicators were 0.87 for *Arm Elevation*; 0.93 for *Shoulder Movement* and 0.81 to *Comfort in Rest*. As for GV, the indicator was low (0.49).

#### Scale reliability indexes

Test-retest reliability index measured by ICC was 0.84 (CI95% of 0.68 to 0.93) for *Arm Elevation* dimension; 0.89 (CI95% of 0.77 to 0.95) for *Shoulder Movement* dimension; 0.94 (CI95% of 0.87 to 0.97) for *Comfort in Rest* dimension and 0.84 (CI95% of 0.68 to 0.93) for GV.

#### Construct validity

**Figure 2:**
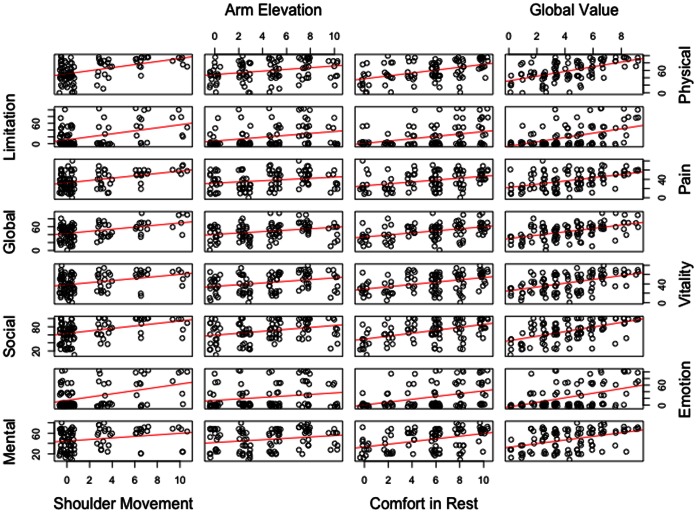
Correlation of Simple Shoulder Test Brazilian-Portuguese Version to SF-36.

The Simple Shoulder Test dimensions presented statistically significant correlation coefficients in the expected directions with all the SF-36 subscales (Figure 2). For *Arm Elevation* dimension, the correlations varied from weak (r = 0.28) to moderate (r = 0.31), with strongest correlations with Limitations through Physical Aspects (r = 0.34), Global Health (r = 0.35), Vitality (r = 0.32) and Social Aspects (r = 0.315). *Shoulder Movement* dimension showed significant moderate correlations with all the SF-36 dimensions (r >0.40), except to Vitality (r = 0.37) and Mental Health (r = 0.25). Correlations coefficients from *Comfort in Rest* dimension ranged from 0.36 to 0.48 for all the SF-36 dimensions, with higher values for Physical Function (r = 0.48) and Social Aspects (r = 0.47). GV had all correlations with a moderate size for all SF-36 variables (r = 0.42 to r = 0.56).

## Discussion

To the best of our knowledge, this is the first study conducting a translation, cultural adaptation, and validation of the Simple Shoulder Test to Brazilian-Portuguese as well as comparing its latent structure among other cultures. The Simple Shoulder Test is an instrument for assessing functional limitations of the shoulder affected by injuries that interfere with an individual’s daily activities [10]. In this study, Simple Shoulder Test scale was translated to Brazilian-Portuguese (Simple Shoulder Test-BR) and it demonstrated adequate internal consistency, reliability and validity to assess patients with shoulder complaints.

Although the Simple Shoulder Test claims to measure a single construct, the factor analysis of the Simple Shoulder Test-BR resulted in three dimensions (*Arm Elevation*, *Shoulder Movement* and *Comfort in Rest*). Adequate fit indicators were obtained based in the parameters suggested by the literature [31], [33], [30]. Roddey et al [7] also obtained more than one dimensional construct for the Simple Shoulder Test with a 2-factor solution. In their study, the first dimension measures what patients can do with their shoulder and the second one also measures the patients comfort with their shoulder at rest. Conversely, when we tested the second order, we observed that SST can have a global value for Shoulder Function, approaching to its one factor solution original structure. So, we could consider SST as a 3-factors solution scale that might be analyzed through a global value representing the shoulder function. Cultural background differences are a possible cause underlying the differences we found across different SST’s factor structures. This difference could be explained by factors such as different interpretations of the same questions or, alternatively, by different professional profiles. For example, since our sample contained subjects whose occupation is frequently related to manual labor, their interpretation of function is likely different from their counterparts in developed countries where most individuals carry their work in an office. Of importance, this hypothesis remains poorly supported as most cross-cultural validations of the SST were not accompanied by a factor analysis.

We observed that the internal consistency of Simple Shoulder Test-BR measured by Cronbach alpha was comparable with the Lithuanian [34], Dutch [8] and Italian [35] versions of Simple Shoulder Test, all of them reporting similar values for Cronbach’s alfa.

In our study, test-retest reliability was satisfactory and was in accordance with the other versions of the questionnaire [34], [8]. Hence, based on previous studies [15], [36] and on our findings, we believe that Simple Shoulder Test-BR has the potential to evaluate functional limitations of an injured shoulder.

To establish construct validity of Simple Shoulder Test-BR, we correlated it to SF-36 questionnaire subscales and found positive correlations. We observed that the three SST-BR (Brazilian-Portuguese version of SST) subscales correlated in the same directions of SF-36 subscales, with strongest correlations in physical aspects. Hence, predicting that the theoretic construct of SST-BR and SF-36 subscales are equivalent. Ryliskis et al [34] also evaluated the construct validity of Lithuanian version of Simple Shoulder Test in comparison with the SF-36 and the Constant Scoring Scale. Mirroring our results, they found positive associations with the SF-36 subscales.

In our study, the Activities of Daily Living (ADL) SST-BR subscale presented a weak correlation with the Mental Health subscale of the SF-36. Oh et al [37] evaluated the measurement properties for the most commonly used shoulder outcome instruments, measuring their association with the SF-36. Similar to our results, most of the evaluated scales were associated with physical components of SF-36, but not with the mental health component.

Matsen et al [38] and Kampen et al [8] also compared Simple Shoulder Test with SF-36 and found that some parameters of health status associated strongly with the patients’ ability to perform different shoulder functions. The Bodily Pain, Physical Function and Physical Role subscales of the SF-36 demonstrated the strongest correlations, which corroborates our findings. Godfrey et al [15] noted a good correlation between Simple Shoulder Test and Physical Functioning Component of SF-12 (r = 0.439) and a strong correlation with American Shoulder and Elbow Surgeons (ASES) shoulder scale (r = 0.807). Therefore, Simple Shoulder Test presents an appropriate construct validity for patients to self-evaluate shoulder function severity and shoulder injury compromising not only their daily activities, but also their quality of life in terms of vitality, general health, and social function.

Romeo et al [13] compared the Simple Shoulder Test with other scales designed to assess shoulder function, namely the UCLA shoulder and the Constant Murley shoulder scales, finding good consistency between them. They also concluded that information for completing the Simple Shoulder Test is easily retrieved and converted into a scale score, facilitating its use in doctors’ offices and in multicenter studies.

One of the limitations of our study is that the Simple Shoulder Test-BR was applied in a single hospital. Although this hospital patient diversity is roughly similar to the average patient population in Brazil, the used sample is not necessarily representative of the entire population with shoulder conditions in Brazil [39].

The Simple Shoulder Test translation and cultural adaptation to Brazilian Portuguese demonstrated adequate validity and reliability and therefore can be used to evaluate shoulder function in patients with shoulder conditions in new multicenter studies. Another possible use of the Simple Shoulder Test translation will be the ability to assess shoulder conditions before and after treatment among Brazilian patients. The translated and validated Simple Shoulder Test questionnaire can finally be used to validate other shoulder scales.

## Supporting Information

Appendix S1 **SST Questionnaire - Original and Brazilian-Portuguese Versions.**
(DOCX)
