# Supplementary material for: Validation of the Simple Shoulder Test in a Portuguese-Brazilian Population. Is the Latent Variable Structure and Validation of the Simple Shoulder Test Stable across Cultures?
Source: PLoS One. 2013 May 13;8(5):e62890. doi: 10.1371/journal.pone.0062890 (PMC3652820; doi:10.1371/journal.pone.0062890)
Supplement: Appendix S1 — SST Questionnaire - Original and Brazilian-Portuguese Versions. (DOCX) [file pone.0062890.s001.docx]

**Appendix**

# SIMPLE SHOULDER TEST

# Dominant Hand (fill in only one oval): Right ( ) Left ( ) Ambidextrous ( )

# Shoulder Evaluated (fill in only one oval): Right ( ) Left ( )

Yes No

# 1. Is your shoulder comfortable with your arm at rest by your side? ( ) ( )

# 2. Does your shoulder allow you to sleep comfortably? ( ) ( )

# 3. Can you reach the small of your back to tuck in your shirt with

# your hand? ( ) ( )

# 4. Can you place your hand behind your head with the elbow

# straight out to the side? ( ) ( )

# 5. Can you place a coin on a shelf at the level of your shoulder without

# bending your elbow? ( ) ( )

# 6. Can you lift one pound (a full pint container) to the level of your

# shoulder without bending your elbow? ( ) ( )

# 7. Can you lift eight pounds (a full gallon container) to the level

# of your shoulder without bending your elbow? ( ) ( )

# 8. Can you carry twenty pounds at your side with the affected

# extremity? ( ) ( )

# 9. Do you think you can toss a softball under-hand twenty yards with

# the affected extremity? ( ) ( )

# 10. Do you think you can toss a softball over-hand twenty yards with

# the affected extremity? ( ) ( )

# 11. Can you wash the back of your opposite shoulder with the affected

# extremity? ( ) ( )

# 12. Would your shoulder allow you to work full-time at your regular job? ( ) ( )

#

# SIMPLE SHOULDER TEST (Brazilian-Portuguese Version)

Mão dominante (escolha somente uma opção): Direita ( ) Esquerda ( ) Ambidestro ( )

Ombro examinado (preencher apenas uma resposta): Direito ( ) Esquerdo ( )

Sim Não

1. Sente seu ombro confortável com seu braço em repouso ao seu lado? ( ) ( )

2. Seu ombro permite que você durma confortavelmente? ( ) ( )

3. Você consegue alcançar suas costas para colocar sua camisa para

dentro da calça com sua mão? ( ) ( )

4. Você consegue colocar a mão atrás da cabeça com o cotovelo bem

para o lado? ( ) ( )

5. Você consegue colocar uma moeda em uma prateleira ao nível do

ombro sem dobrar o cotovelo? ( ) ( )

6. Você consegue levantar meio quilo, ao nível do ombro sem dobrar

o cotovelo? ( ) ( )

7. Você consegue levantar 4 quilos ao nível do ombro sem dobrar

o cotovelo? ( ) ( )

8. Você consegue carregar 10 quilos ao seu lado com o braço que dói? ( ) ( )

9. Você acha que consegue lançar uma bola de tênis por baixo com a mão

do lado afetado a uma distância de 20 metros? ( ) ( )

10. Você acha que consegue lançar uma bola de tênis por cima com a

mão do lado afetado a uma distância de 20 metros? ( ) ( )

11. Você consegue lavar a parte de trás do ombro bom, usando o

lado que dói? ( ) ( )

12. O seu ombro permite que você trabalhe por período integral no

seu trabalho? ( ) ( )
